# Mendelian Randomization analysis of the causal effect of adiposity on hospital costs

**DOI:** 10.1016/j.jhealeco.2020.102300

**Published:** 2020-03

**Authors:** Padraig Dixon, William Hollingworth, Sean Harrison, Neil M. Davies, George Davey Smith

**Affiliations:** a Population Health Sciences, University of Bristol, United Kingdom; b MRC Integrative Epidemiology Unit, University of Bristol, United Kingdom; c NIHR Biomedical Research Centre, University of Bristol, United Kingdom

**Keywords:** BMI, Obesity, Instrumental variables, Healthcare costs, Mendelian Randomization

## Abstract

Estimates of the marginal effect of measures of adiposity such as body mass index (BMI) on healthcare costs are important for the formulation and evaluation of policies targeting adverse weight profiles. Most estimates of this association are affected by endogeneity bias. We use a novel identification strategy exploiting Mendelian Randomization – random germline genetic variation modelled using instrumental variables – to identify the causal effect of BMI on inpatient hospital costs. Using data on over 300,000 individuals, the effect size per person per marginal unit of BMI per year varied according to specification, including £21.22 (95% confidence interval (CI): £14.35-£28.07) for conventional inverse variance weighted models to £18.85 (95% CI: £9.05-£28.65) for penalized weighted median models. Effect sizes from Mendelian Randomization models were larger in most cases than non-instrumental variable multivariable adjusted estimates (£13.47, 95% CI: £12.51-£14.43). There was little evidence of non-linearity. Within-family estimates, intended to address dynastic biases, were imprecise.

## 1 Introduction

A positive association between adiposity and healthcare costs is well established. It has been documented for a variety of different contexts, circumstances and health systems (Cawley, 2015; Finkelstein and Yang, 2011; Withrow and Alter, 2011). This association has powerful economic salience because of its apparent consequences for the level, growth and composition of healthcare spending.

The underlying biological relationship between adiposity and health is complex (Corbin and Timpson, 2016), but the endocrinal (Corbin et al., 2016), cardiometabolic (Emdin et al., 2017; Lyall et al., 2017) and other changes (Wang et al., 2011) associated with increased adiposity are themselves linked to substantial healthcare resource requirements (Lehnert et al., 2013). These demands on healthcare resources have arisen in a wider context in which increases in the mean and variance of adiposity, reflected in widely used measures of nutritional status such as body mass index (BMI - weight divided by the square of standing height) have led to important changes in the global distribution of adiposity (Davey Smith, 2016; Finucane et al., 2011; N. C. D. Risk Factor Collaboration, 2016). The worldwide prevalence of overweight (BMI> = 25 kg/m^2^) and obesity (BMI> = 30 kg/m^2^) is 28.8% for men and 29.8% for women. This accounts for some 2.1 billion individuals, an increase of approximately 50% since 1980 (Ng et al., 2014). More individuals globally are now either overweight or obese than are underweight (Black et al., 2013; N. C. D. Risk Factor Collaboration, 2016).

Correlational evidence of the BMI-cost association is influential. Examples of this influence include the development of guidelines and policies to prevent obesity (Government Office for Science, 2007), evaluation of interventions targeting overweight and obesity (Avenell et al., 2004), and the prioritization of research into the consequences of obesity (Kraak et al., 2005). However, a critical limitation of much if not all of this multivariable or conditional correlational^1^ research is that it can be seriously affected by endogeneity bias (Auld and Grootendorst, 2011).

This endogeneity arises through three channels. The first is measurement error arising from mismeasurement of BMI (and other measures of adiposity), particularly where individuals self-report weight (Burkhauser and Cawley, 2008; Cawley et al., 2015a). The second is reverse causation or simultaneity bias, which would occur if healthcare costs influenced adiposity. The third source of bias is omitted variable bias, arising from unknown or unmeasured common causes of both adiposity and healthcare costs.

The direction of the omitted variable bias will generally not be known a priori. Disease processes that are related to healthcare costs may also influence adiposity. For example, higher BMI is associated with increased risk for cancers (Lauby-Secretan et al., 2016), but cancer (including prodromal cancer) may itself lead to reductions in BMI (Tisdale, 2002). Similarly, people with higher BMI are more likely to smoke, while smoking itself lowers BMI (Carreras-Torres et al., 2018; Taylor et al., 2018). Without evidence of the wider determinants of both adiposity and healthcare cost, the analyst cannot reliably predict directions of bias when undertaking multivariable analyses of this association.

BMI-health outcome associations are therefore distorted because one of the drivers of healthcare costs is own health status. Reliable evidence of the causal association between adiposity and healthcare costs is a critical input for the formulation and evaluation of cost-effective policies and interventions targeting (in particular) overweight and obesity (Wang et al., 2011), as well as for identifying research priorities in this area. The widespread use of models lacking robust identification may substantially under- or over-estimate the true causal effects of BMI.

This observation has motivated attempts to use instrumental variable (IV) analyses in which the instrument for own BMI is the BMI of a biological relative, for example in relation to the association between BMI and mortality (Davey Smith et al., 2009). This approach has also been used to model the causal impact of adiposity on costs, and arguably represents the most credible attempt to date to overcome the endogeneity biases of conventional multivariable analysis.

For example, Cawley and Meyerhoefer (Cawley and Meyerhoefer, 2012) used the BMI of a biological relative as an IV. This suggested that the healthcare costs of obesity were drastically underestimated by prior multivariable conditional correlational analyses, with a fourfold difference in the marginal costs of obesity between multivariable and causal IV analysis reported, and a threefold difference in the costs of a marginal unit of BMI. Large but less pronounced differences between multivariable and IV models were also reported in studies using similar instruments by Black et al. (2018b), Cawley et al. (2015b), Doherty et al. (2017) and Kinge and Morris (2018).

However, this approach does have limitations. The association of biological relatives and healthcare costs may itself be affected by omitted variables that are common and independent causes of both BMI and healthcare costs. These could include the home environment that is shared by biological relatives and which may influence food consumption, proclivity to exercise, and access to and use of healthcare services. People who have children (necessary for the biological relative approach) may differ from those who do not have children. Intrauterine influences of maternal BMI on offspring BMI, such as smoking and alcohol drinking during pregnancy (Lawlor et al., 2017), and genetic influences that affect healthcare costs other than through adiposity (Dixon et al., 2016), will also confound this relationship.

This paper exploits a novel identifying approach - germline genetic variation associated with BMI – in an instrumental variable analysis. This approach has the advantage (in principle) of avoiding the limitations of both multivariable conditional correlational analysis and the use of a biological relative as an instrument.

At each point of variation in the genome, offspring inherit one allele from their mother, and one from their father. An allele is the specific adenine (A), cytosine (C), guanine (G) or thymine (T) nucleobase that is inherited at each point of variation in the genome. This inheritance of alleles is a natural experiment, in which individuals in a population can be divided into groups based on their inherited “dose” of these variants (Evans and Davey Smith, 2015). If the instrumental variable assumptions hold, these genetic variants can be used to test whether BMI affects healthcare costs.

Using genetic variants as IVs in this way has become known as Mendelian Randomization (Davey Smith and Ebrahim, 2003). Very large, high-quality datasets that can facilitate this type of analysis are beginning to become available (Collins, 2012; Sudlow et al., 2015) but remain largely if not entirely unexploited by health economists studying the causal effect of health conditions and traits on cost outcomes. Our results indicate that our base point effect estimates (obtained from inverse variance weighted Mendelian Randomization models) for a marginal unit of BMI £21.22 (95% confidence interval (CI): £14.35-£28.07) are approximately 57% larger than non-instrumental variable multivariable adjusted estimates (£13.47, 95% CI: £12.51-£14.43). The Mendelian Randomization effect estimates attenuate somewhat under sensitivity analyses that are robust to violations of the exclusion restriction. For example, estimates from the penalized weighted median model (£18.85 (95% CI: £9.05-£28.65)) are lower than the inverse variance weighted estimate but still higher than the conventional non-instrumental variable point effect.

Below, we set out the broad context of our analysis by first reviewing how the biology of genetic variation and conditionally random allocation of genetic variation at conception might be used as instrumental variables in general. We relate how the general principles of Mendelian Randomization operate in the specific empirical context of our research question. We then present our approach to estimation and sensitivity analysis, in which we test, as rigorously as possible, assumptions that are both general to instrumental variable analysis and those that are specific to Mendelian Randomization. This latter analysis has a particular focus on assessing the impact of heterogeneity at the level of genetic variants.

These methods are applied to data from the UK Biobank, an exceptionally large, detailed and high-quality genotyped dataset that is linked to the universe of publicly funded inpatient hospital care episodes. We interpret our results in relation to their policy implications, having regard to the limitations of this analysis.

## 2 Methods

### 2.1 Mendelian Randomization and instrumental variable analysis

Here, we briefly introduce the high-level biological mechanisms that motivate the use of genetic variants in IV analysis. More detailed introductions and extended overviews of Mendelian Randomization are available elsewhere (Davey Smith and Hemani, 2014; Davies et al., 2018; Pingault et al., 2018; von Hinke et al., 2016).

A single nucleotide polymorphism (SNP) is a specific location (or locus) in the human genome that differs between people in the population. At each SNP people will have two alleles, one for each chromosome. During cell division at conception (meiosis), offspring inherit at random one of their mother’s two alleles, and one of their father’s two alleles. Specific SNPs or sets of SNPs are known to associate with particular health conditions or to influence the development of particular traits. Thus, the phenotype (a measurable disease or trait such as BMI) may be influenced by genotype (an underlying genetic structure associated with the phenotype).

The provenance of the term Mendelian Randomization (Davey Smith, 2007), and the potential utility of genetic variants as IVs, is founded on Mendel’s first and second laws of inheritance. The first law describes random segregation of alleles from parent to child during the formation of gametes. The second law describes the independent assortment of alleles for different phenotypes at conception. Genetic variants that are in different locations in the genome are generally inherited in a way that is independent of the inheritance of other genetic variants. The allocation of these genetic variants to offspring is therefore random, conditional on parental genotype.

We now describe the core instrumental variable assumptions in the context of Mendelian Randomization. These assumptions can be described as comprising the relevance assumption, the independence assumption, and the exclusion restriction. In what follows we refer interchangeably to SNPs, variants and genetic variants.

The first IV assumption (“relevance”) is that the instrument should be associated with the treatment variable, which in this paper is BMI.^2^ Some of the initial IV studies using genetic variation in economics (e.g. (Ding et al., 2009; Fletcher and Lehrer, 2011; Norton and Han, 2008)) relied on evidence of relevance obtained from so-called “candidate gene” studies, which proceed from an assumed, anticipated or measured relationship between particular regions of the genome and a phenotype of interest. Relevance can be threatened in these circumstances, since these associations were often not robustly tested and candidate gene associations have been observed to have poor replicability (Benjamin et al., 2011, 2012; Chabris et al., 2015; Ioannidis et al., 2011). As could have been anticipated (Colhoun et al., 2003), Fletcher (Fletcher, 2018) notes of this early economics literature that “Indeed, in hindsight, one might expect that none of the results reported in papers using candidate gene approaches are robust.”

We anticipate that the relevance assumption will be readily met in our study since we do not use a candidate gene approach. The associations of SNPs with diseases and traits are in general better determined from genome wide association studies (Hirschhorn and Daly, 2005; McCarthy et al., 2008), which study the independent association with specific phenotypes of many SNPs - potentially millions - across the genome. These associations are corrected for multiple testing so that genome-wide significance is obtained as the conventional p-value threshold value based on an alpha of 0.05 divided by *k*, where *k* can be interpreted (conservatively) as the number of independent statistical tests conducted across the genome (Bush and Moore, 2012). These associations will be validated in independent replication samples. Following convention, we will describe p< = 5 × 10^−8^ as genome-wide significant. We describe the genome wide association studies that we use in the Data section.

The second assumption is that there are no omitted variables in the associations of the IV and the outcome (healthcare costs). This assumption is plausible since SNPs are determined at conception, and therefore prior to the postnatal circumstances, events and behaviours of later life. However, time of conception (such as month or year of birth) could theoretically associate with SNPs and healthcare costs. Population stratification, the separation of individuals into distinct subgroups that differ in allele frequencies, is another means by which the second assumption may be violated, since differences in alleles in this case would indicate differential ancestry rather than disease susceptibility (Cardon and Palmer, 2003).

Ancestry influences the distribution of genetic variants, but also risks of disease not necessarily attributable to those variants. This potential confounding by ancestry is typically accounted for by adjusting for the genetic principal components and restricting analysis to genetically homogenous ethnic groups. Fletcher (Fletcher, 2018) further notes that failure to control for population structure is also likely to have affected early economic studies using Mendelian Randomization.

This can introduce bias induced by spurious associations. A notable example of this is provided by Rietveld et al. (Rietveld et al., 2014) as follows. Genome wide association studies of educational attainment would find associations between education and a gene for lactase persistence, because both educational attainment and lactase persistence vary by ancestry. However, lactase persistence is almost certainly unrelated to cognitive influences on educational attainment. This spurious association remained after restricting analysis samples according to self-reported ethnicity, but was removed when adjustments were made for genetic principal components.

Simultaneity bias, if present at all and absent population stratification, is likely to be modest in the case of adiposity and healthcare costs. Examples of the independence of common genetic variation from common omitted variables (and thus that SNPs are likely to be independent of environmental influences in support of this assumption) has been demonstrated empirically (Davey Smith and Hemani, 2014; Davey Smith et al., 2007).

The third IV assumption is that the SNP(s) affect the outcome only via the treatment variable; that is, via the condition or trait of interest. This is the exclusion restriction. Violations of this assumption are the primary threat to the validity of IVs used in Mendelian Randomization. There are two principal mechanisms by which this assumption may be violated in Mendelian Randomization.

The first is the correlation of the SNP(s) in question with other SNPs that affect the outcome through a path other than via the condition or trait of interest (Lawlor et al., 2008). This correlation of variants, known as linkage disequilibrium, arises when particular variants tend to be inherited together (contrary to Mendel’s second law), generally because they are located in close physical proximity on the genome (Visscher et al., 2012).

The second mechanism concerns variants that affect more than one phenotype through independent pathways (Cawley et al., 2011; Davey Smith and Hemani, 2014; Hemani et al., 2018a). A SNP that affects BMI may also, for example, affect the risk of depression through a BMI–independent mechanism. IV analysis relating, for example, a set of BMI SNPs to healthcare cost outcomes would suffer from bias in this case if depression independently affects both BMI and healthcare costs. This is sometimes known as horizontal pleiotropy (Davey Smith and Hemani, 2014). Pleiotropy (Paaby and Rockman, 2013; Stearns, 2010) is the effect of a single SNP on multiple phenotypes (Lobo, 2008; Stearns, 2010). There would be no bias in this analysis if depression was on the causal pathway between BMI and healthcare costs, a situation sometimes referred to as vertical pleiotropy (Davey Smith and Hemani, 2014), or if the other phenotype did not affect the outcome of interest.

Our starting position is that horizontal pleiotropy, in violation of the exclusion restriction, is likely to be present amongst some of the SNPs that we study. This is both because pleiotropy appears to be pervasive throughout the human genome (Hemani et al., 2017), and because of the outcome that we study. Our outcome, hospital costs, can be influenced by anything that is associated with hospital attendance. This may open horizontal pleiotropic pathways that may not be observed when studying other economic outcomes.

For example, both excess adiposity and depression may influence hospitalization, whereas either of these phenotypes may or may not influence performance on a different outcome such as an academic test of intelligence. Our response to the possibility of pleiotropy is to test for its presence, and to implement pleiotropy-robust estimators as described below. Our work represents an early application of many of these methods in the health economics literature.

We now consider monotonicity. Monotonicity requires that the direction of effect on the treatment from varying the level of the instrumental variable should be in the same direction for all individuals. When monotonicity is satisfied, IV analysis (including Mendelian Randomization) identifies a local average treatment effect; that is, an effect in those whose treatment would differ if the value of the IV differed. This is the average effect of BMI on hospital costs for individuals whose BMI was affected by the 79 BMI increasing SNPs. Mendelian Randomization therefore does not identify population average treatment effects.

The consequence of the monotonicity assumption is that for all individuals at all 79 SNPs, replacing an allele associated with lower BMI with an allele increasing BMI would either increase BMI or leave their BMI unchanged. Monotonicity may be biologically plausible (Burgess and Thompson, 2015a) but cannot be demonstrated empirically, since to do so would require a comparison of the consequence for BMI of replacing an individual’s observed BMI associated genotypes with a counterfactual alternative genotype.

In conventional instrumental variable models, compliers (individuals who satisfy these assumptions) are a subgroup of our entire analysis sample but generally cannot be identified. This subgroup may be equivalent to the entire analysis sample, or may constitute a large or small subgroup of that sample. Note that because BMI is continuous variable, it is reasonable to consider the LATEs as reflecting the impact of the SNPs across the entire distribution of BMI. This would suggest that the complier subgroup could comprise the entirety of our sample. Thus, it is plausible that the average treatment effect is equivalent to the LATE since the effects of BMI on compliers are likely to be similar to the effects of BMI on average across the wider population.

In any event, there is no evidence from the Locke et al. GWAS (Locke et al., 2015) on which we rely that BMI-SNP associations have the opposite sign (violating monotonicity) in any of the subgroups studied in that GWAS. We conduct our analysis and report its findings under the assumption that monotonicity holds for most if not all of our analysis sample. I.e. that the local average treatment effect identified by our estimator is equal to the average effect in the population.

Finally, that all of the preceding assumptions may be met in a particular example does not guarantee that reliable causal inferences can be obtained if the instrumental variables are weak. Weak instruments are another of the issues identified by Fletcher (Fletcher, 2018) as affecting earlier economic literature, despite recognition in initial economics discussions of Mendelian Randomization (Fletcher, 2011; von Hinke Kessler Scholder et al., 2011) and in more recent analyses (Davies et al., 2015; von Hinke et al., 2016) of the importance of strong instruments. Even if SNPs satisfy the relevance assumption at genome-wide levels of significance, it is possible that they are “weak” instruments, in the sense of explaining only a small proportion of the variance in the treatment in any given finite sample (Burgess et al., 2015; Davies et al., 2015). Weak instruments will bias the causal estimate in finite samples toward the non-IV estimate in one sample models and toward the null in two-sample Mendelian Randomization models (Burgess and Thompson, 2015a; Davies et al., 2015) and will affect the estimate precision in all cases (von Hinke et al., 2013).

Our approach to instrument strength is a point of departure from the earlier Mendelian Randomization economics literature. We report the proportion of variance explained in BMI by our instruments, compare our base case estimates to a method robust (under assumptions) to weak instruments, and we use some of the largest samples studied to answer this research question. We estimate our base models on over 300,000 unrelated individuals in population-wide analysis, and over 28,000 related individuals for our within-family analysis.

### 2.2 Model estimation

For a single SNP, the ratio (or Wald) estimator can be calculated as the ratio of the SNP-outcome to the SNP-treatment variable (BMI) associations. This gives the effect of the variant on the outcome, scaled by the effect to the SNPs on the treatment. This is equivalent to the two-stage least squares estimator for a single SNP. Using the terminology of Bowden et al. (2015), indexing individuals by *i* and denoting SNPs as *G* (indexed *j* from 1 up to *J*) these two relationships can be written as:

(1) $$X=\sum_{j=1}^{J}{\gamma_{j}G}_{ij}+\varepsilon_{j}^{X}$$

(2) $$Y=\sum_{j=1}^{J}\alpha_{j}G_{ij}+\beta X_{i}+\varepsilon_{i}^{Y}$$

Without loss of generality, we ignore constants and exogenous omitted variables in Eqs. (1) and (2). The alpha term is the direct effect of variants on the outcome that do not operate through the BMI treatment variable. If the exclusion restriction holds then alpha will be zero, since valid instruments influence outcomes only through an effect on the treatment.

Note also that the two associations described in Eqs. (1) and (2) in the Wald estimator need not come from the same sample, in which case a two-sample IV estimator is used (Angrist and Krueger, 1992). A two-sample approach using summarized data may offer similar or better efficiency than a single sample study using individual-level data, particularly if larger sample sizes are available under a two-sample approach. In the two-sample setting, genetic variants must have similar effects in each population (Haycock et al., 2016).

Rewriting Eqs. (1) and (2) into the reduced form, and using $\varepsilon_{i}^{`Y}$ to denote the error term, yields:

(3) $$Y={\text{Γ}}_{j}G_{ij}+\varepsilon_{i}^{`Y}=(\alpha_{j}+\beta \gamma_{j}{)G}_{ij}+\varepsilon_{i}^{`Y}$$

The ratio estimate is ratio of the effect of the SNPs on the outcome (${\text{Γ}}_{j}$), scaled by their effect on the treatment ${(\gamma }_{j})$, which can be written (ignoring the error term) as:

(4) $$\frac{{\text{Γ}}_{j}}{\gamma_{j}}=\frac{\alpha_{j}+\beta \gamma_{j}}{\gamma_{j}}=\frac{\alpha_{j}}{\gamma_{j}}+\beta$$

The ratio estimates from each individual variant can be combined using weighted regression or equivalently inverse variance weighted (IVW) meta-analysis to produce an overall causal estimate (henceforth for simplicity we refer to this estimate as the IVW estimate – Eq. (5)). This assumes that there no correlation between the Wald estimates for each SNP, which will hold if they are not in linkage disequilibrium.

(5) $${\hat{\beta }}_{IVW=}\frac{\sum_{j=1}^{J}{\hat{\gamma }}_{j}^{2}\sigma_{Y_{j}}^{-2}{\hat{\beta }}_{j}}{\sum_{j=1}^{J}{\hat{\gamma }}_{j}^{2}\sigma_{Y_{j}}^{-2}}$$

Here, the $\sigma_{Y_{j}}^{-2}$ terms are the variance of the error term in the outcome-SNP regression models; the small variance of the error term in the treatment-SNP regression is ignored (the no-measurement error assumption).

If the exclusion restriction holds, there should be no more heterogeneity in the estimates for all SNP effect sizes than would be expected by chance. This can be assessed using Cochran’s Q statistic (Cochran, 1950; Higgins and Thompson, 2002) in two-sample settings (this is closely related to Sargan’s over-identification test (Sargan, 1958)), which follows a ${\text{χ}}^{2}$ distribution with *J*-1 degrees of freedom:

(6) $$Q=\sum_{j=1}^{J}\frac{1}{\sigma_{Y_{j}}^{2}}({{\hat{\beta }}_{j}-\hat{\beta }}_{IVW})$$

Cochran’s Q can identify failure of the instrumental variable assumptions, but not whether this is due to one, some or all IVs being invalid, or why they are invalid. As such, it is a relatively crude “catch all” test of instrument validity. Nevertheless, it is useful as a first step to indicate the presence of heterogeneous causal effects across the instrument set, which may be due to pleiotropy, but potentially also other violations of the instrumental variable assumptions.

### 2.3 Sensitivity analysis

A number of methods have been developed to accommodate violations of the exclusion restriction due to pleiotropy that is suggested by (but not necessarily unambiguously identified by) high heterogeneity as determined by Cochran’s Q (Davey Smith et al., 2018; Hemani et al., 2018a). The following considers some of these methods, which follow the spirit of Conley et al. (2010) in relaxing the assumption that the $\alpha_{j}$ parameter of Eq. (4) is exactly zero. The underlying goal is to apply methods to generate consistent estimates of the causal effect even if some or all of the IVs are invalid.

If pleiotropy (i.e. non-zero $\alpha_{j}$ terms) is present but small in magnitude, then biases in any causal analysis will be modest. If $\alpha_{j}$ is zero on average across all SNPs then the relationship is estimated with more noise and hence some loss of efficiency than if all $\alpha_{j}$ values were zero, but the bias term will have zero mean on average even if some or all of the pleiotropic effects are large. In this case, the IVW estimator could be implemented using a random effects meta-analysis.

If the mean effect of alpha is not zero, then directional pleiotropy is present. So-called MR-Egger methods allow for directional pleiotropy by modelling both the slope and intercept of the ratio estimator of Eq. (4).

(7) $${\hat{\text{Γ}}}_{j}=\beta_{0}+\hat{\gamma_{j}}\beta_{1}+\varepsilon_{i}^{Y}$$

Note that the “$\hat{\text{Γ}}$, $\hat{\gamma_{j}}$” terms included in Eq. (7) are themselves estimates, respectively of the SNP-cost and SNP-BMI estimates. MR-Egger estimators are less powerful and less efficient than the estimators discussed below because of the need to estimate both the slope parameter and the intercept parameter.

All SNPs can be invalid instruments under MR-Egger, provided that the InSIDE (Instrument Strength Independent of Direct Effect) assumption holds. The MR-Egger effect estimate can be written as $\frac{cov\left({\hat{\text{Γ}}}_{j},{\hat{\gamma }}_{j}\right)}{var({\hat{\gamma }}_{j})}$ which can be re-expressed as the true effect estimate $\hat{\beta }$ plus a bias term $\frac{cov\left(\hat{\alpha },{\hat{\gamma }}_{j}\right)}{var({\hat{\gamma }}_{j})}$. The bias term will be zero when the numerator is zero – that is, when instrument strength (${\hat{\gamma }}_{j}$) is independent of the direct effect ($\hat{\alpha }$) of the SNPs on the outcome.

This is the InSIDE assumption, and appears to be plausible in some cases (e.g. (Pickrell et al., 2016)) but not in others (e.g. (Bowden et al., 2015; Davey Smith and Hemani, 2014)). The assumption will hold in circumstances where genetic associations with other variables are uncorrelated with each other (Pickrell et al., 2016) (Burgess and Thompson, 2017). It may also hold when pleiotropy is horizontal, which would rule out a direct effect of a variant contributing to instrument strength.

The InSIDE assumption can be violated if, following (Burgess and Thompson, 2017), SNPs influence omitted variables in the BMI-outcome association. For example, consider SNPS that have horizontal pleiotropic effects on some health condition that is such an omitted variable. In this case, there will be a positive correlation between these pleiotropic effects and instrument strength, since instrument strength will be inflated by the influence of the pleiotropic variants. This will induce covariance between strength and the direct effect of pleiotropic variants, in violation of the InSIDE assumption. In general, SNPs with larger effects on these omitted variables will tend to have larger pleiotropic effects and higher instrument strengths. Burgess and Thompson (Burgess and Thompson, 2017) conclude “It is difficult to imagine how the InSIDE assumption could be satisfied if several genetic variants have pleiotropic effects acting via confounders.”

An alternative to relying on the InSIDE assumption is to use the median ratio estimate of all available instruments (Bowden et al., 2016). This estimator will be unbiased if more than half of the instruments are valid, i.e. $\alpha_{j}=0$ for at least half of all SNPs. The simple intuition for this estimator is that invalid instruments in the IVW approach will contribute weight to the overall regression estimate and will be biased even asymptotically. On the assumption that the majority of instruments are valid, then invalid instruments contribute no weight and are less biased than IVW in finite samples and unbiased asymptotically. We implement a penalized weighted median estimator. SNPs contributing to the median 50% of the statistical weight are used to form the median estimate. The weights are a function of the precision with which SNPs are estimated in the Locke et al. (2015) genome wide association study, and the penalization involves “down weighting” outlying SNPs that contribute substantial heterogeneity to the Q statistic.

The final class of estimators we consider are mode based (Hartwig et al., 2017). The underlying assumption, in terms of the alpha expression, is that $Mode\left(\alpha_{1},\alpha_{2},\ldots \alpha_{k}\right)=0$. The intuition is that classifying variants into clusters based on similarity of effect will be consistent if the largest homogenous cluster are valid SNPs. All other SNPs outside this cluster, even a majority of SNPs, could be invalid, provided this “zero modal pleiotropy” assumption holds. This approach requires the setting of an arbitrary bandwidth parameter to define the clusters. We implement a more efficient version of the simple mode estimator by weighting median estimates by the inverse variance of the effect of the SNPs on the outcome. This is given effect by creating an empirical density function formed from the weighted mode estimates.

It is important to note that the second and third IV assumptions are not directly testable, and the assumptions underlying alternative modelling approaches for $\alpha_{j}$ term are themselves untestable. However, these approaches are important forms of sensitivity analysis that allow the instrumental variable assumptions to be relaxed, albeit at the cost of other untestable assumptions. Similarity of estimated effect under each of the estimators considered would offer some reassurance that the same causal effect is being identified, although it is important to note that MR-Egger is much less precise than other estimators.

It is also important to note the Mendelian Randomization is most robust within the family unit, since genetic differences between pairs of siblings necessarily reflect independent, random meiotic events. This point was made in the first extended systematic formulation of the notion, in 2003 (Davey Smith and Ebrahim, 2003): “The basis of Mendelian randomization is most clearly seen in parent–offspring designs that study the way phenotype and alleles co-segregate during transmission from parents to offspring…. Thus the Mendelian randomization in genetic association studies is approximate, rather than absolute”. It was also made as far back as RA Fisher’s articulation (Fisher, 2010) of the logic of statistical genetics in 1951: “The different genotypes possible from the same mating have been beautifully randomised by the meiotic process. A more perfect control of conditions is scarcely possible, than that of different genotypes appearing in the same litter.”

The central issue then becomes whether the approximation is “good enough” for the purposes of making reliable inferences under population-wide MR. On the one hand, the independence of allele scores from large numbers of non-genetic potential confounding variables has been demonstrated (e.g. (Davey Smith et al., 2007; von Hinke Kessler Scholder et al., 2014)), which suggests that random allocation of alleles at the level of the parent-child relationship during conception holds, conditional on population structure, outside of the family trio. Using a single individual from each family should not suffer from omitted variables that would otherwise confound the relationship between treatment variables and economic outcomes (von Hinke et al., 2016).

On the other hand, the increasingly large scale of genetic data sources is beginning to reveal patterns of association with environmental confounds, including patterns of geographic settlement within otherwise apparently homogenous ethnic populations, that cannot be eliminated or “adjusted away” by using the types of control for population structure that were implemented in our analysis models (Haworth et al., 2019). This is potentially problematic; in the specific example of geographical similarity since cultural, economic and other factors may also differ by location and could constitute omitted variables that confound the associations we study.

An important analysis alongside our “population”-based main analysis is therefore that of within-family analysis – we provide more details below. This was complemented by a range of other sensitivity analyses. The first considered whether the association between BMI and healthcare cost may be non-linear, the second estimated a multivariable Mendelian Randomization instrumenting for both BMI and body fat percentage, the third assessed whether results were robust to potential weak instrument bias, the fourth examined a gene-environment interaction as a means of identifying and correcting for pleiotropy (Chen et al., 2008; Davey Smith, 2010; Spiller et al., 2018), and the fifth considered a disaggregation of the cost outcome.

#### 2.3.1 Within-family Mendelian Randomization

Within-family Mendelian Randomization is intended to address biases from dynastic effects (Brumpton et al., 2019; Fletcher, 2011), but may also avoid biases caused by cryptic population structure not accounted for by restricting analysis to homogenous ethnic groups and the use of genetic principal components. Dynastic effects refer (in the present context) to the direct effect of parents’ BMI on their children. This type of effect may reflect non-transmitted alleles – even if children do not receive a BMI-increasing SNP, their parents may possess such a SNP and this in turn can influence the environment in their children are raised. If present, the main Mendelian Randomization analysis presented here would wrongly attribute some of the influence of parental BMI to the child’s BMI-increasing SNPs that are included in the analysis. We therefore explored whether bias from dynastic effects could be reduced by conducting a within-family Mendelian Randomization in which a family “fixed effect” adjusts for environmental conditions created by parents that are shared by offspring (Pingault et al., 2018).

Siblings were identified in the UK Biobank by using data on kinship taken from the KING toolset and data on the proportion of loci shared between individuals. More details are available in Brumpton et al. (2019). We restricted analysis to the IVW estimator. This is because the MR-Egger, median and mode estimators used in the main analysis on the sample of unrelated individuals have lower power than the IVW estimator. The sample of included related individuals is less than 10% (n = 28,608) of that used in the main analysis, and the statistical power of IVW methods is therefore much reduced.

We estimated fixed effect instrumental variable models, clustering on family units, and conditioning on sex. Family fixed effects control for time-invariant unobservable characteristics that are specific to each family. We compared the results of these models to those obtained from within-family models that excluded the family fixed effect but clustered standard errors at the level of the family.

#### 2.3.2 Non-linear models

There is some evidence of a non-linear association between BMI and hospital costs from multivariable and causal studies (e.g. (Cawley and Meyerhoefer, 2012; Cawley et al., 2015b)). Fitting non-linear models in the IV settings is complicated when the instruments explain a relatively small proportion of variance in the treatment (as in the present example), because any non-linear effects may not be detectable in the relatively narrow range over which such effects influence the treatment-outcome association (Staley and Burgess, 2017b). This can be seen from comparing mean BMI in individuals with the lowest decile of the allele score (26.3 kg/m^2^) to those in the highest decile (28.5 kg/m^2^).

To avoid this, we used methods developed by Staley and Burgess (Staley and Burgess, 2017a). This approach proceeds from the observation that stratifying on the BMI distribution by dividing it into categories or quantiles over which the non-linearity can be assessed would violate the exclusion restriction. This is because the premise of IV analysis is (in the present context) that BMI is an intermediate step on the causal pathway between the IV and the cost outcome. BMI is therefore a potential outcome of both the IV and of the outcome, since hospitalisation itself may affect BMI, and naïve stratification on BMI would represent over-adjustment by inducing an association between the IV and the outcome in violation of the exclusion restriction.

The starting point for this analysis was therefore to calculate residual BMI. Residual BMI is the difference between BMI and the fitted values obtained from a regression of BMI on the weighted allele score. Residual BMI for an individual is therefore predicted BMI for a (hypothetical) individual with no BMI-increasing alleles. The study cohort was then divided into 100 quantiles of residual BMI.

The (linear) Mendelian Randomization approaches described above were then conducted within each quantile, to give quantile-specific “local average causal effect” estimates. These can be interpreted as the average change in costs, within that quantile of the residual BMI distribution, for a one-unit increase in genetically predicted BMI. This results in 100 local average causal effects. These effects were combined into a plot of local average causal effects for 100 quantiles to compared against the corresponding quantiles of the original BMI distribution.

Meta-regression was then applied to these effect estimates. Meta regression, in this context, assesses the extent to which differences or heterogeneity between these local average causal effects can be related to differences in BMI. Absence of heterogeneity would indicate similarity of the causal effect of BMI on costs across the distribution of BMI and provide evidence in support of linearity. Meta regression was implemented by estimating fractional polynomial models of degrees 1 and 2. This permitted a fractional polynomial test, which tests whether linear or non-linear models offer a better fit to the data. A trend test was also used, which regressed the local average causal effect estimate on mean BMI in each quantile.

#### 2.3.3 Multivariable Mendelian Randomization – BMI and body fat

Multivariable Mendelian Randomization can estimate the direct causal effect of more than one treatment variable (Burgess et al., 2014; Burgess and Thompson, 2015b). In this application of multivariable Mendelian Randomization, genetic variants for BMI and for percentage of body fat were included in the same instrumental variable model. This allows for these biologically related treatments to be modelled together, and for the potential mediation of one treatment (BMI for example) by another (body fat percentage) to affect the outcome. The coefficients in the estimated models reflect the direct causal effect of each treatment, holding the other treatment fixed. These models have considerably lower power to detect causal effects than univariable Mendelian Randomization, but the analysis can nevertheless usefully estimate the direct effect of BMI on outcome compared to the total (comprising the direct effect of BMI and its indirect effects via body fat percentage) estimated in conventional Mendelian Randomization (Sanderson et al., 2018). For the purposes of comparison, we also estimated models utilizing percentage of body fat only.

For this analysis we remain agnostic as to which of the two measures of adiposity that we study below- BMI and percentage of body fat – more accurately index the health-compromising consequences of fatness. The percentage of body fat arguably better captures body composition than does BMI and may better predict particular health outcomes (e.g.(Burkhauser and Cawley, 2008; Kragelund and Omland, 2005; Yusuf et al., 2005)), but BMI nevertheless retains broad applicability and utility as an easily measured variable that offers robust associations with a variety of relevant health outcomes (Corbin and Timpson, 2016).

#### 2.3.4 Weak instruments

We estimated the “robust adjusted profile score” model of Zhao et al. (2018), which is unbiased in the presence of many weak instruments, and is also robust to measurement error in SNP-treatment estimates. The Zhao et al. approach relies on a version of the InSIDE assumption that underpins the MR-Egger approach, but unlike MR-Egger assumes that the pleiotropic effects $\alpha_{1},\alpha_{2},\ldots \alpha_{k}$ have mean zero.

Our use of this model is conservative, since we use only genome-wide significant SNPs from the Locke et al. (2015) BMI genome wide association study. Nevertheless, we report it as an additional sensitivity analysis. If our instruments are weak, we would expect to observe a large difference in the causal estimate between the robust adjusted profile score and our base inverse variance weighted estimates.

#### 2.3.5 Gene-by-environment interaction

We considered gene-by-environment interactions as a means of detecting and correcting for pleiotropy. If an instrument (such as a BMI SNP or set of SNPs included in an allele score as described below in Section 3.3) interacted with a covariate induces variation in the association between the instrument and the BMI treatment variable, it is possible to identify and correct for pleiotropic effects. This approach builds on work (Cho et al., 2015) (Slichter, 2014) that considers a no-relevance population subgroup for which instrument and treatment variables are independent.

In the context of Mendelian Randomization, an instrument-outcome association for a no-relevance subgroup would indicate the presence and extent of pleiotropy, which could then be subject to bias correction. This is because SNPs that are valid IVs can only influence the healthcare cost outcome by their effect on BMI. This approach is set out in Spiller et al. (2018), which does not depend on the existence of an observed no-relevance subgroup, and in essence places the IV assumptions on the interaction between the IV and the covariate, rather than solely on the IV.

We modelled an interaction between the BMI IV and socio-economic deprivation, and separately between the BMI IV and participant age. Deprivation reflects access to material goods (such as car ownership), occupational status and education level. We note that, for both the BMI IV-deprivation and BMI IV-age estimates to be valid, it is necessary to assume that pleiotropic effects do not differ between the population subgroups.

#### 2.3.6 Type of outcome

We also assessed whether any heterogeneity present in the main analysis was also present when disaggregating overall inpatient hospital costs into elective costs, non-elective costs, and other costs. More details on the definitions of these terms and the analysis undertaken is provided in supplementary material.

## 3 Data

### 3.1 UK biobank

Individual-level data were drawn from the UK Biobank study. This very large, high quality prospective cohort enrolled 503,317 adults (representing a response rate of approximately 5.5%) aged between 37 and 73 (99.5% of enrollees were aged between 40 and 69) living in England, Scotland and Wales (Fry et al., 2017). Invitations to participate were issued to all eligible adults. However, participation itself was not random, with the consequence that the Biobank cohort is not representative of the wider population from which it is drawn. In particular, the cohort is healthier (Sudlow et al., 2015) (lower levels of mortality and lower rates of morbidity-increasing behaviour such as smoking) and is better educated (Fry et al., 2017) than the wider UK population.

At the baseline appointment, participants completed a number of questionnaires, biomarker specimens were drawn, physical function was assessed, and consent was given to link these data to death registers and healthcare records (Sudlow et al., 2015). Deprivation was calculated using the Townsend Deprivation Index and divided into quintiles when treated as a covariate in the Spiller et al. (2018) gene-by-environment sensitivity analysis described above.

Weight and height were measured at the baseline appointment by nurses. Weight was measured using weighing devices. Body composition was measured using bio-impedance (opposition of alternating current to adipose tissue). Both measures were very similar (Lin’s rho p-value <0.001) and impedance-based BMI data were used when the conventional BMI data were missing. Observations that had a mean difference between traditional and impedance-based measures of BMI of more than 5 standard deviations from the mean difference were excluded from the analysis. Whole body percentage fat mass calculated from impedance measurements was used in the multivariable Mendelian Randomization analysis.

### 3.2 Measurement of costs

The hospital care that we measure was provided by hospitals operating under the aegis of the National Health System (NHS) in England and in Wales. The NHS is a taxpayer funded provider of universal healthcare. In principle, there are no relevant differences in access by eligible individuals to NHS services by region or other characteristics. In practice, there may be modest differences in access to hospital care, such as slightly longer waiting times for treatment in some areas, but these are unlikely to be relevant to the methods or conclusions of this study.

The hospital costs that we analyze are those borne by the NHS as a public provider of universal health care. The data we access is not a sample of hospital care episodes; instead it is a census that captures the universe of all inpatient care in these hospitals. This encompasses both publicly funded care provided in NHS and private hospitals, as well as privately funded care (arranged through private health insurance, for example) that is performed in the public hospital system. Thus, the coverage of hospital costs accounts for all publicly funded care but does not include care in private hospitals that is not arranged and funded by the NHS, data for which is not available for the UK Biobank cohort.

Admitted patient care episodes, sometimes referred to as inpatient care episodes, were obtained from Hospital Episode Statistics (HES) (for English care providers) and from the Patient Episode Database for Wales (for Welsh providers) that were linked to UK Biobank. Inpatients are those admitted to hospital and who occupy a hospital bed but do not necessarily stay overnight (i.e. day case care). Due to differences in the collection and valuation of care in Scottish hospitals compared to hospitals in England and Wales, only costs from the latter two jurisdictions are included in this analysis. Linkages to other forms of care were not available at the time of writing.

Each “Finished Consultant Episode” (FCE) on inpatient care can be characterized by a number of variables, most importantly procedure codes and diagnosis codes (based on ICD-10 codes (World Health Organization, 1992)). These FCEs were converted, using NHS software (NHS, 2016), into Healthcare Resource Groups (HRGs). HRGs are used for casemix-adjusted remuneration of publicly-funded hospitals in England and Wales. Unit costs were assigned to each HRG, and inpatient costs per person year of follow-up were calculated for each patient on the basis of their recorded FCEs (if any). Further details on the cost calculations are given in Dixon et al. (2018).

Only episodes and UK Biobank baseline appointments occurring on or after 1 April 2006 were eligible to be included in the analysis because of changes to the hospital payment system that came into effect at that time (Department of Health, 2012). Data on inpatient episodes was available until patient death, patient emigration (rates of which are estimated to be a modest 0.3% (Fry et al., 2017)), or the censoring date for inpatient care data of 31 March 2015. Cost data are reported in 2016/17 pounds sterling.

Hospital cost data is often skewed and individuals who did not report hospital use have no hospital costs. Despite these features being present in our hospital data, we used the Mendelian Randomization estimators (inverse variance weighted, MR-Egger, penalized weighted median and weighted mode) described above to analyze these data for two reasons. The first is that instrumental variable models still produce a policy-relevant average causal effect estimate of the association between BMI and healthcare costs even if this association is non-linear.

The second argument, based on Zhao et al. (2018), is that SNPs explain a modest proportion of the variance in the outcome via the treatment variable. In our case, any difference induced by the SNPs in the BMI treatment variable therefore only requires the assumption of linearity over a small range, and this assumption will hold to a satisfactory approximation whenever the function is differentiable over that range – the full logic behind this claim is set out in supplementary material.

We can use this logic to map out the shape of the relationship by examining these associations over quantiles of the BMI distribution. We can use techniques for non-linear instrumental variable analysis to make claims about the similarity or otherwise of the causal effect of BMI on healthcare costs at different quantiles of the BMI distribution (Staley and Burgess, 2017a). This is the same set of techniques that we use model and test for non-linear effects as described above.

### 3.3 Genetic data and linkage to phenotypic data

Genetic data was subject to quality controls by UK Biobank, as well as further in-house processing and management (Harrison, 2019; Mitchell et al., 2017). Briefly, 488,377 individuals in the UK Biobank were successfully genotyped. Removal of individuals was performed as follows: sex mismatches and individuals with abnormal numbers of sex chromosomes, related individuals, and those who withdrew consent. To avoid biases from population stratification, the sample was restricted to individuals of white British ancestry (as determined by self-report or analysis of genetic principal components (Bycroft et al., 2018)). Bringing together all the genetic and phenotypic data, including the cost data necessary to calculate IV models, resulted in 307,048 individuals included in the analysis. Further detail on these steps is provided in the Supplementary Material. Related individuals were analyzed separately for the within-family Mendelian Randomization analysis.

The most recent and largest genome-wide association study of BMI that did not explicitly overlap (Yengo et al., 2018) (i.e. include individuals who appeared in both the genome-wide association analysis sample and the UK Biobank sample) was Locke et al. (2015). Proxy SNPs were used for any SNPs identified in Locke et al. (2015) but not present in UK Biobank, provided that a suitable proxy with an R^2^ statistic between the proxy and missing SNPs of at least 0.8 was available in UK Biobank. To avoid violations of the IV assumptions due to linkage disequilibrium, only SNPs that were correlated with each other with an R^2^ of less than 0.001 within 10,000 kilobases were retained for analysis using the MR-Base R package (Hemani et al., 2018b). In total, 79 of the 97 genome-wide significant SNPs identified in Locke et al. (2015) were included in the analysis, following this process and the removal of triallelic and unreconciled palindromic SNPs. SNP data were harmonized between Locke et al. and UK Biobank so that each source corresponds to the same allele.

Locke et al. includes groups of heterogenous ancestry (Berg et al., 2018). The list of 79 SNPs from Locke et al. included those from studies of both European and non-European ancestry. In sensitivity analysis, we re-ran the Mendelian Randomization analysis restricting the SNPs (n = 69) from Locke et al. that were discovered using individuals of European ancestry only. The restriction of the set of SNPs to those identified as genome-wide significant in the Locke et al. GWAS was intended to test the sensitivity of the results to greater homogeneity between the two samples used, at the cost of a possible reduction in power. Power may be lower because of the smaller number of SNPs used, and a possible reduction in the proportion of variance in BMI that these SNPs explain. However, power would not have reduced relative to the base case if these SNPs only affected people of non-European origin. Data on SNPs implicated in fat mass percentage used in multivariable analysis were taken from Lu et al. (2016).

Both the individual variants and a summary polygenic allele score created from these variants were used in analysis. The allele score was used in tests of association between potential omitted variables present at conception that were available in UK Biobank (sex, year of birth, month of birth) using linear regression. The allele score was calculated as the sum of the BMI-increasing alleles for SNPs attaining genome wide significance in Locke et al. (2015). Each SNP was weighted by the size of its effect on BMI.

We compared the Mendelian Randomization estimates to those from multivariable conditional correlational models by estimating the effect of a marginal unit of BMI on costs using ordinary least squares models and a generalized linear model with a gamma family and log link function following Dixon et al. (2018).^3^ In these models we controlled for sex, days of exercise, frequency of alcohol consumption, educational qualifications, employment status, quintiles of deprivation, and age at recruitment to the UK Biobank cohort. We assume for both the OLS models and the generalized linear models that none of these controls are potential outcomes of both BMI and of the cost outcome.

The causal estimates from the Egger, median and mode estimators were converted from standard deviation units of BMI reported in the Locke et al. (2015) to natural units of BMI by dividing by the median standard deviation of BMI (4.6) in that study, as reported in Budu-Aggrey et al. (2018). This rescaling allows the results of all estimators to be interpreted as the marginal effect of a unit (kg/m^2^) increase in BMI on inpatient costs.

Analysis was conducted primarily in R using the MR Base package (Hemani et al., 2018b). Stata version 15.1 (StataCorp, College Station, Texas) was used for some elements of the analysis. Analysis code is available at github.com/pdixon-econ

## 4 Results

Of the 307,048 individuals included in the analysis sample, 54% were female (n = 164,903), and mean age was 56.9 years (standard deviation: 8.0). Mean BMI was 27.4 kg/m^2^ (a histogram of BMI is provided in supplementary material). Some 55% (n = 168,486) of patients had positive inpatient hospital costs. Mean and median follow-up of inpatient hospital data was 6.1 years. The most common ICD-10 chapters under which patients were admitted (other than for symptoms and findings not otherwise classified) were neoplasms (most commonly breast cancer) and musculoskeletal disorders (most commonly arthropathies).

Mean inpatient hospital cost per person-year of follow-up was £479, while median costs were £88. There was evidence of association of the BMI allele score with nine of the first ten principal components (largest p-value, from the eighth principal component = 0.11)) and weaker evidence of association with month (p = 0.46), year of birth (p = 0.07) and sex (p = 0.06). Sex and all ten principal components were included as covariates in all Mendelian Randomization models. The F-statistic from the first stage of a two-stage least squares model using the BMI allele score as an instrumental variable was 697, and the same statistic was measured as 96 when including all 79 SNPs as individual instrumental variables in the same type of model.

Results indicate that the effect of an additional unit of BMI is approximately 58% higher using IVW methods than under multivariable generalized linear analysis and 48% higher than the ordinary least squares estimate (Table 1).

**Table 1:** Mendelian Randomization and multivariable estimates of marginal effect of an additional unit of BMI on per person year inpatient hospital costs.

	Beta (£)	SE	P Value
**Estimator**			
Inverse variance weighted random effects estimator (IVW RE)	21.22	3.50	<0.001
Multivariable generalized linear model estimator	13.47	0.49	<0.001
Ordinary least squares estimator	14.35	0.52	<0.001

*Note*: We report p-values smaller than 0.001 as <0.001. Larger p-values are reported to two decimal places.

**Fig. 1:**
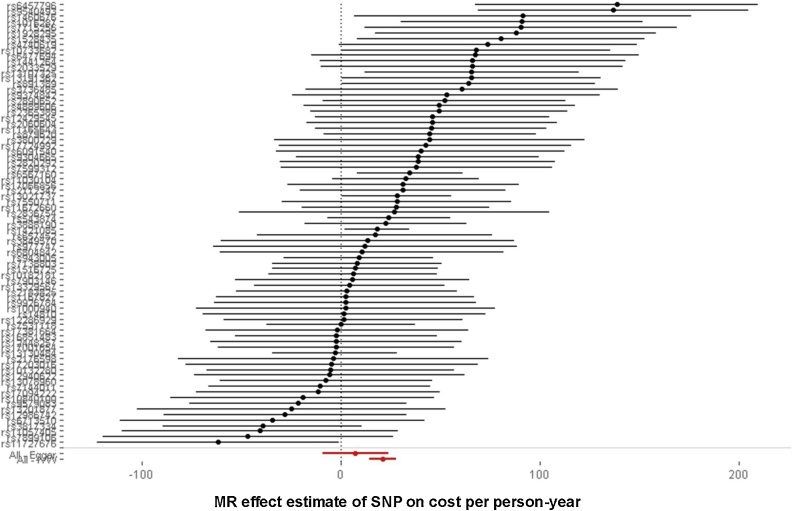
Forest plot of SNPs. *Note*: This table lists effect sizes and 95% confidence intervals for all 79 SNPs, ordered according to positive effect size on the outcome. The two diamonds at the bottom of the plot represent the IVW estimate from using all SNPs (“All – IVW”) together with a 95% confidence interval, and also for contrast the MR-Egger estimate (“All – Egger”) with a 95% confidence interval when using all 79 SNPs. Point estimates are positive in each case, but MR-Egger is associated with much greater uncertainty that the IVW estimate.

However, there is evidence of heterogeneity (Cochran’s Q = 107.8, p-value for null of no heterogeneity = 0.01) in the base IVW results, one cause of which may be pleiotropy in violation of the exclusion restriction. Heterogeneity is apparent in the forest plot (Fig. 1). A forest plot without heterogeneity would show all variants “lining up” around the same point estimate of effect, subject to sampling variation which will mean that not all variants would lie on precisely the same line.

The results of MR-Egger and other methods to adjust for pleiotropy are indicated in Table 2, presented for comparison alongside the base IVW results.

**Table 2:** Results of primary Mendelian Randomization models.

	Beta (£)	SE	P-value
**Estimator**			
IVW RE (for reference)	21.22	3.50	<0.001
MR-Egger	7.41	8.44	0.38
Penalized weighted median	18.85	5.00	<0.001
Weighted mode	16.75	6.08	0.01

*Note*: We report p-values smaller than 0.001 as <0.001. Larger p-values are reported to two decimal places.

**Fig. 2:**
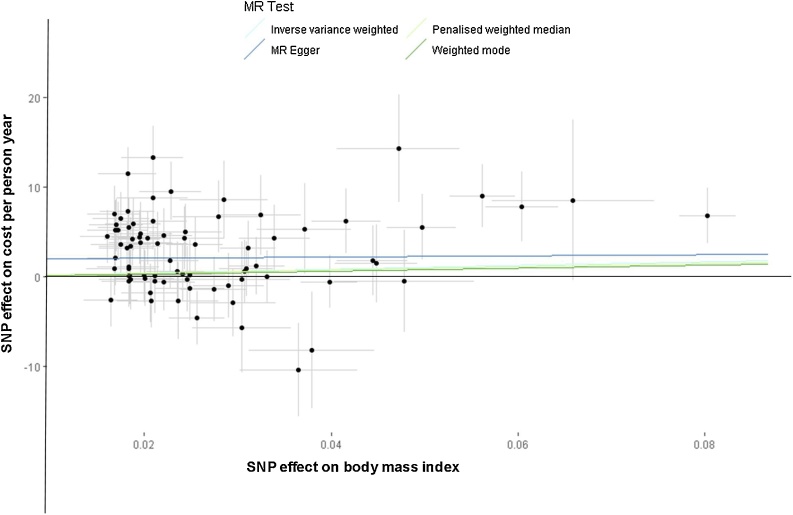
Scatter plot and effect estimates for four main estimators. *Note*: All 79 SNPs are plotted together with 95% confidence intervals representing their effect on both BMI (horizontal access) and on the cost outcome (vertical axis).

All estimators identify a positive effect of BMI on hospital costs, although the MR-Egger estimates are imprecise. Fig. 2 presents a scatter plot summarising the results from the four estimators presented in Table 2. Results from models that also conditioned on age were similar – details are provided in supplementary material.

The MR-Egger Cochran’s Q test of heterogeneity was 103.44 (p-value 0.02) and the intercept of this model was estimated as £1.93 (standard error: 1.07, p-value: 0.08). The IVW effect estimate is larger than all other estimates, although similar to the penalized weighted median estimate. If pleiotropy is present in the IVW model but not in the penalized weighted median model, it appears to be inflating the effect estimates, which would be the case if some of the included SNPs act on other conditions or traits that tend to increase inpatient costs on average.

The InSIDE assumption, which must be satisfied for MR-Egger estimates to be unbiased, is most likely to hold where the violations of the IV assumptions are caused by pleiotropy that does not influence omitted variables in the BMI-cost association. The rationale for this position was discussed in the Methods section above. In practice, there is probably good reason to suspect violations of this type, as any variant that influences, for example, mental health may well be an omitted variable that independently influences both BMI and inpatient costs. In the case of this hypothetical example, instrument strength (measured by the association of BMI) may be correlated with a direct effect of the SNP (via mental health) on the cost outcome. Thus, any SNP included amongst the 79 here that causes people to have inpatient care may well induce violations of InSIDE.

It is notable that the median and mode estimators are reasonably similar, despite the differences in the assumptions underlying each method. This is suggestive evidence that a similar causal effect is perhaps being identified by these two methods.

**Fig. 3:**
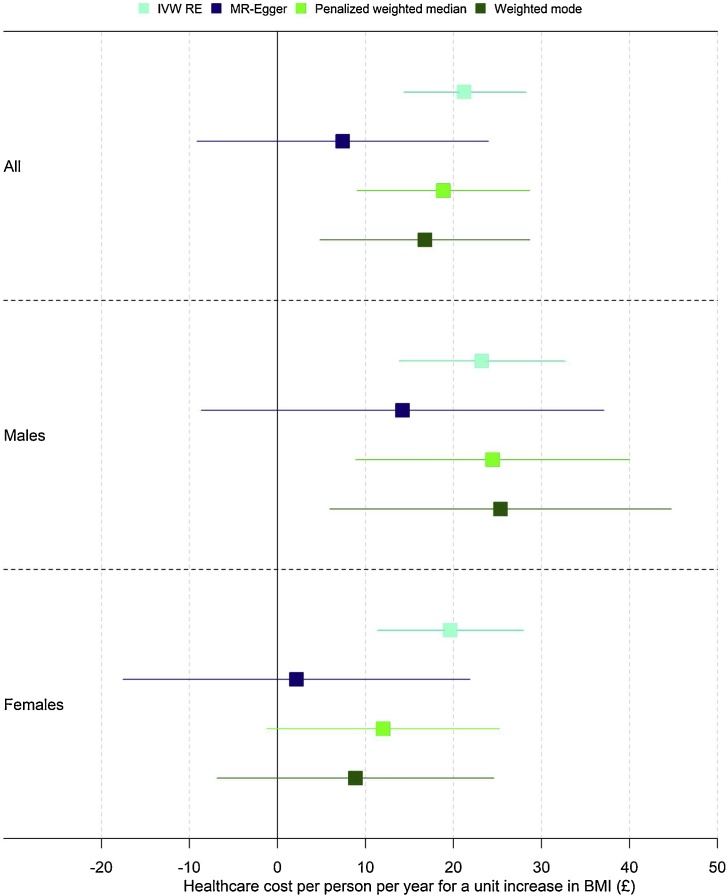
Mendelian Randomization results overall and by sex. *Note*: Each dot represents a point estimate, together with a 95% confidence interval, corresponding to the results in Table 2, Table 3.

Evidence for heterogeneity is less apparent when stratifying on sex (Table 3). Evidence of heterogeneity was weak when estimating separate models for men and women (Cochran’s Q: males: Q = 82.71, p-value = 0.33; females: Q = 92.05, p-value = 0.13.) Effect sizes were larger for males than for females, although confidence intervals overlapped for all estimators (Fig. 3).

**Table 3:** Mendelian Randomization results by sex.

	Beta (£)	SE	P-value
**Estimator**			
*Males*			
IVW RE	23.21	4.78	<0.001
MR-Egger	14.21	11.67	0.23
Penalized weighted median	24.45	7.94	<0.001
Weighted mode	25.34	9.89	0.01
*Females*			
IVW RE	19.64	4.16	<0.001
MR-Egger	2.16	10.05	0.83
Penalized weighted median	12.01	6.72	0.07
Weighted mode	8.86	8.02	0.27

*Note*: We report p-values smaller than 0.001 as <0.001. Larger p-values are reported to two decimal places.

For the within-family analysis, 28,608 individuals were observed in 13,838 family units.

The estimated effect of an additional unit of BMI was £16.42 (standard error 19.10, p-value 0.39) in within-family Mendelian Randomization models controlling for sex (but not genetic principal components). The effect size estimated is similar but slightly smaller than in all other analyses but is very imprecisely estimated.

The same model estimated without family fixed effects, controlling for sex again without genetic principal components) and clustering standard errors at the family level was also consistent with the null with a similar effect size (£11.96, standard error 10.38, p-value = 0.25). Both estimates are similar to each other in having point estimates lower than the base case estimates, and are both very imprecisely estimated, with standard errors similar to the absolute size of the point estimate.

These results differ from our main analysis in somewhat smaller effect sizes, which may indicate that dynastic biases are inflating estimates in our population wide “unrelated” sample used for the main analysis. However, these results are also very imprecise, and power to reject the null in this sample is weak, given that the sample used in the main analysis is more than ten times larger, and that the included SNPs happen to explain less of the variation in BMI (see below). This suggests null results from the within-family models may be a false negative associated with weak statistical power in the within-family sample. Evidence from Kong et al. (2018) suggests that the effect size of non-transmitted BMI-increasing alleles is smaller than the effect size for transmitted alleles (as modelled in the main analysis), which is another consideration to suggest that dynastic effects may not be a large source of bias in this context.

Differences between the within-family and unrelated samples also complicate interpretation of our findings. The within-family sample differed from the sample of unrelated individuals in relation to the proportion of females (57.8% versus 53.5%), mean cost (£600 versus £479) and mean age at recruitment (57.6 versus 56.9 years); p < 0.001 for the null of no difference for all of these comparisons. The relationship of the 79 SNPs to BMI also differed between samples – these SNPs explained 1.74% of the variance in the unrelated sample but only 1.64% in the within-family sample.

However, mean BMI (p = 0.07) and mean BMI allele score (p = 0.45) were similar between the within-family sample and the sample of unrelated individuals. There was no difference in the size of the absolute effect of the SNPs on BMI in any sample, whether measuring this as a within-family effect, treating siblings as individuals, or on the sample of unrelated individuals. The estimated effect of the allele score on BMI was close to 4.0 in each specification (i.e. each unit increase in the allele score increases BMI by 4 units), obtained using simple linear regression models controlling for sex, except for the within-family estimates which were implemented using a fixed-effects model.

Along with dynastic biases, we also cannot completely rule out other explanations for our findings, especially those that would give rise to a non-random distribution of alleles in the population, due to assortative mating or residual population stratification. We consider the meaning and implications of these terms below in the Discussion.

Overall, larger sample sizes, potentially involving meta-analysis across cohorts where within-family Mendelian Randomization is possible, would provide the best means to definitively understand whether statistical power, differences between within-family and unrelated samples, or substantive dynastic or other biases explain our results from within-family models. We cannot eliminate dynastic effects as a possible source of bias in our analysis.

### 4.1 Other sensitivity analyses

Using the full base case sample of unrelated individuals, there was little evidence of non-linearity. There was evidence consistent with the null for a quadratic term (p = 0.88), for differences in local average treatment effect estimates across quantiles (p = 0.15), for heterogeneity in the associations between the instrument and BMI across quantiles (p = 0.26) but some evidence of a linear trend in the association between the instrument and BMI across quantiles (p = 0.03). We conclude that the association between adiposity and inpatient hospital costs for this sample is approximately linear. This association is presented graphically in the supplementary material.

There was modest attenuation of the effect of BMI on costs when including body fat percentage in a multivariable Mendelian Randomization analysis. The causal coefficient on the body fat percentage IV was consistent with the null, while the effect estimate on BMI was within the confidence intervals of the base IVW estimate (Table 4).

**Table 4:** Results of multivariable Mendelian Randomization analysis.

	Beta (£)	SE	P-value
**Genetic variants**			
IVW RE of total effect of BMI only (for reference)	21.22	3.50	<0.001
BMI	22.40	9.12	0.01
Body fat percentage	−2.75	13.31	0.84

*Note*: We report p-values smaller than 0.001 as <0.001. Larger p-values are reported to two decimal places.

In a Mendelian Randomization analysis using body fat percentage only the IVW effect estimate was consistent with null (£10. 76, SE 8.15, p-value = 0.18) per additional percentage point of body fat. However, there was evidence of heterogeneity (Q = 32.58, p-value<0.001). MR-Egger estimates were also null (£18.14, SE 40.17, p-value = 0.66), but the other pleiotropy-robust estimates suggested point estimates for a one percent increase in body fat that were broadly similar to (if slightly higher than) those estimated for an additional unit of BMI. The penalized weighted median estimate was (£22.05, SE 6.97, p-value = 0.001) and the weighted mode estimate (£23.11, SE 8.18, p-value = 0.02).

Overall, this suggests that any direct effect of body fat percentage on hospital costs is limited, and body fat percentage probably does not mediate the effects of body mass index on hospital costs. If body fat percentage were a mediator, the causal effect of BMI would change much more markedly between the conventional and multivariable Mendelian Randomization analyses. Pleiotropy-robust estimates for both BMI and body fat percentage indicated a causal effect on hospital costs that were or roughly similar magnitude, albeit body fat percentage estimates were somewhat higher.

Application of the robust adjusted profile score method of Zhao et al. (2018) (to assess possible impacts of weak instrument bias) did not substantially alter the base IVW estimates of the causal effect, estimating a causal effect per additional unit of BMI of £21.69 (standard error 3.06, p < 0.001) compared to the base IVW estimate of £21.22 (standard error 3.50, p < 0.001). Subject to the assumptions of the method, particularly that all pleiotropic effects have mean zero, this suggests that weak instruments and measurement error in the SNP-treatment association are not likely to be material sources of bias, at least for the base case results.

The results of the gene-by-environment test, in which the BMI allele score was interacted with deprivation as a means of detecting and correcting for pleiotropy, were imprecise. Although the Spiller et al. (2018) method did identify a positive effect of BMI on healthcare costs (£12.69, standard error 5.44), this estimate was consistent with the null (p-value = 0.10). Note also that these estimates lie within the confidence interval for the MR-Egger estimates, which may reflect a lack of precision to identify a robust directional pleiotropic effect in these two analyses. The null result for the gene-by-environment test may also reflect a violation of the constant pleiotropy assumption in this sample, in which the magnitude of the pleiotropic effect is not the same across levels of deprivation. Imprecise results were also obtained when interacting the allele score with quintiles of age, although the magnitude of the effect differed in size and sign (-£12.13, standard error = 6.59, p-value = 0.16)

Models using a set of SNPs derived from using SNPs genome-wide significant for those only of European ancestry indicated lower effects sizes and greater differences between the median and mode estimators (Table 5). These models have somewhat lower power than the base models, but this may not explain more than a small part of the attenuation of effects observed.

**Table 5:** Results of all Mendelian Randomization models with restricted SNP list.

	Beta (£)	SE	P-value
**Estimator**			
IVW RE for reference	18.70	3.80	<0.001
MR-Egger	6.47	11.02	0.56
Penalized weighted median	16.10	5.03	0.001
Weighted mode	7.48	8.41	0.38

Heterogeneity was also somewhat lower when using the restricted list of SNPs, with Cochran’s Q for the IVW model of 88.32 (p-value = 0.04). This suggests that pleiotropy (amongst other reasons) may be less important for these results than for the main results, and it is notable that the IVW RE estimates become closer to those of the pleiotropy-robust base case estimates. This may also explain the divergence between the penalized weighted median and weighted mode estimates that is apparent in Table 5, although we have no way of testing this.

Finally, we considered disaggregation of all costs into elective costs, non-elective costs and other costs. Detailed results are provided in supplementary material. The largest absolute effect of BMI appears to be on elective care costs, for which estimated heterogeneity (as measured by Cochran’s Q) was similar to that for overall aggregate costs. While suggestive, caution is required in interpreting these results. First, the categorizations used are somewhat arbitrary. Second, comparing the disaggregated costs both to each other and to all costs involves comparing different groups of individuals, since some cohort members report costs only in one subcategory of costs.

## 5 Discussion

The long-established positive association between adiposity and hospital costs appears to be causal. The results presented here using a novel Mendelian Randomization methodology suggest that this effect of a marginal unit of BMI is higher than that suggested by conventional multivariable analyses.

Below, we consider the choice of a preferred model of all of those estimated. We then compare our findings to the literature, consider the generalizability of these findings, discuss potential remaining biases and conclude with an assessment of the policy relevance of our work.

### 5.1 The choice of a preferred model

We start our discussion of a preferred set of estimates with consideration of statistical power. Given the complexity of BMI as a trait (many SNPs contribute to BMI), and given that effect sizes of individual SNPs are modest at best, very large sample sizes are required to obtain sufficient statistical power to avoid the risks of (a) falsely failing to reject a null hypothesis (b) overstating effect sizes when the null is not rejected.

Some of the estimators had relatively low power to reject the null hypothesis. The MR-Egger regression had lower power than the other population-based methods (as opposed to within-family methods) because it estimates twice the number of parameters than these other methods in estimating both an intercept (which is used to test and adjust for any pleiotropy) and the slope estimate (which measures the combined causal effect of all SNPs). The Spiller gene-by-environment method (Spiller et al., 2018) was also imprecise; this approach has lower power than other methods (in part) because it estimates an interaction term.

Our within-family analysis may be affected by low statistical power. In addition to the within-family fixed effect models, the sibling sample without family fixed effects was also imprecise.

Imprecise estimates from our within-family analysis represent an important contextual finding for the interpretation of our main results but do not oblige us to discard or discount the results from the population-wide Mendelian Randomization estimators.

Estimates derived from well powered sources – inverse variance weighted estimators, median-based estimators, and mode-based estimator – rejected the null, and suggested that the BMI has causal effects on hospital costs. Taking this into account, and given the evidence of pleiotropy that may have affected the inverse variance weighted estimators, we conclude that the penalised weighted median and the weighted mode estimators are, potentially, the most reliable guides to the effect of the marginal unit of adiposity on hospital costs in the sample of unrelated individuals. However, larger within-family models would offer the most compelling source of evidence.

### 5.2 Comparison with other findings

Estimated differences between IV and adjusted multivariable models are smaller than those obtained from analyses using biological relatives as instruments, albeit these other studies were conducted on samples that may differ quite markedly from the sample studied here. Studies by Black et al. (2018a) on Australian data, Kinge and Morris (2018) on UK data and Doherty et al. (2017) on Irish data are studies on children and adolescents. The Black et al. (2018a) study specifically excludes inpatient costs. Doherty et al. (2017) measure resource utilization in primary care and inpatient hospital stays rather than healthcare costs. Cawley et al Cawley et al. (2015b) specifically focuses on BMI impacts in relation to diabetic status. Nevertheless, all studies find that conventional research designs and estimators (such as OLS) suggest lower effect sizes than those from instrumental variable models. This is also a feature of our Mendelian Randomization estimates.

The study with the most similar outcome measure for an adult population is probably that of Cawley and Meyerhoefer (Cawley and Meyerhoefer, 2012), who provide estimates of the effect of the marginal unit of BMI on inpatient hospital costs in their US sample ($54, 2005 price levels). The average closing price of US dollars to sterling during 2005 was 1.82 dollars to one pound sterling (Macrotrends, 2019), making the marginal unit in 2005 in the Cawley and Meyerhoefer sample worth approximately £29.67, or £36.51 in 2016/17 pounds sterling (adjusting for inflation using the GDP deflator (HM Treasury, 2019)). This figure is drawn from a different health system with a different population to UK Biobank (being less selected and likely to be more representative of the underlying population) but nevertheless does indicate a very roughly comparable magnitude of effect if one accounts for higher healthcare costs in the US relative to the UK.

The above studies examine the effect of adiposity in different populations and contexts, but generally find a larger difference between multivariable conditional correlation estimates and IV models, in contrast to the smaller relative difference found in our study. One possible explanation for a larger difference in effect sizes between these types of estimators is attenuation bias, caused by measurement error in the relative effect of BMI, which would tend to inflate differences between multivariable and IV analysis, since the multivariable results may be biased downward. UK Biobank estimates are based on high-quality independent (i.e. not self-reported) measurements of weight and height, whereas models relying on self-report BMI may exhibit more attenuation bias and thus exaggerate the difference between IV and non-IV results. This is speculative but may explain some of the difference in relative effect sizes between our study and other findings in the literature.

More generally, there is a lack of a “gold standard” against which to judge multivariable and IV models or the various Mendelian Randomization estimators. Methods are being developed to choose amongst MR estimators including machine learning (Hemani et al., 2017) and principled approaches to the treatment of “outlier” SNPs (Cho et al., 2019), although a degree of judgement and some contextual reasoning seems unavoidable in interpreting Mendelian Randomization analysis.

Despite the absence of a clear means to choose between types of estimator, there seems to be grounds to argue that policy evaluations and other quantitative analysis requiring estimates of the marginal cost of a unit of BMI should treat multivariable conditional correlation estimates as a lower bound. Analysts should consider including higher estimates of the cost of a marginal BMI unit in primary empirical analysis and undertake sensitivity analysis that tests the robustness of conclusions to lower estimates.

### 5.3 Generalizability of findings

Are the results from this analysis likely to be generalizable to wider populations? Two issues merit consideration. The first is whether the Mendelian Randomization estimates are themselves helpful in understanding the effect of BMI on inpatient hospital costs. The second is whether the particular features of the UK Biobank sample, which is healthier and wealthier than the population from which it is drawn (because of non-random participation), may itself create bias. We consider policy relevance separately below.

On the first point, Mendelian Randomization methods estimate, in this case, the effects on inpatient costs of a lifelong exposure to BMI-increasing SNPs, rather than a temporary or acute effect of higher or lower BMI. We use the term “lifelong” (Holmes and Smith, 2017) to refer to the effect of genetic variation determined at conception and assume that the association between the genetic variants and the relative effect of BMI does not change with age. The effect sizes estimated under all but the MR-Egger Mendelian Randomization analyses were larger in magnitude than the multivariable estimates, which suggests that they may reflect a cumulative exposure to higher BMI (Holmes et al., 2017).

It is plausible that lifelong exposure to higher BMI, randomly determined at conception, could manifest in higher rates of inpatient admission and the use of more complex and expensive treatments amongst the middle-aged and early-old aged individuals represented in the UK Biobank cohort. As BMI is potentially modifiable, this suggests that policies targeting reductions in BMI (where clinically appropriate to do so) could reduce use of hospital resources (amongst other impacts on morbidity and mortality (Wade et al., 2018)).

The second issue concerning the generalizability of our findings relates to the similarity or otherwise of the UK Biobank cohort to the wider population, and the implications that any differences may have on the generalizability of the results presented here. Relative to the UK population, participants in the cohort study had lower levels of mortality (Sudlow et al., 2015), lower rates of health-compromising behaviour, and are better educated (Fry et al., 2017). BMI and use of hospital resources may themselves influence participation in the study (since sicker individuals were less likely to participate), and some degree of selection bias is possible (Hughes et al., 2018b). This specific bias goes by different names, including “collider bias” (Munafò et al., 2017; Spirtes et al., 2000) and bias due to “bad controls” (Angrist and Pischke, 2009).

This selection appears to be problematic (in terms of bias and Type 1 error rates) for Mendelian Randomization only when selection effects are themselves particularly large (Hughes et al., 2018a). Since the size of this effect will generally be unknown (because the mechanism driving selection is unknown) it is not possible to be definitive about its scope in the present context. Gkatzionis and Burgess (Gkatzionis and Burgess, 2018) suggest, on the basis of their simulations, that selection in general is probably less important as a source of bias than, for example, violations of the exclusion restriction caused by pleiotropy. It is also important to note that selection will also affect the non-causal multivariable estimates of a marginal unit of BMI presented alongside the causal IV analysis. It is possible that the precise figure for a marginal unit of BMI under either method may differ in other cohorts but nevertheless the ratio of the causal to non-causal costs will be stable when studied in similar settings.

### 5.4 Potential remaining biases

Three potential remaining sources of bias may be present in our main analysis due to assortative mating, cryptic population structure, and cohort effects.

Assortative mating refers to departures from random mating (Vandenberg, 1972) and may affect our analysis of unrelated individuals. The simulation and modelling study of Hartwig et al. (2018) found that bias from assortative mating would affect all forms of Mendelian Randomization analysis described above, including methods that attempt to account for pleiotropic SNPs. Bias from assortative mating can overestimate SNPs-BMI and SNP-inpatient costs associations. This bias is larger when the strength of non-random assortment is high, the outcome is highly heritable and when the process of non-random mating has been present for a number of generations. In the absence of data relating to these influences, we simply note that this bias may be present to some extent in the results presented here for population-wide analysis but not for sibling models, and that data on family trios (parents and offspring) would help assess if assortative mating was present.

Second, Mendelian Randomization analysis may be confounded by cryptic geographic or population structure. There is some evidence, for example, that geographic structure is present in the UK Biobank sample (Haworth et al., 2019), which would re-introduce bias due to, for example, environmental omitted variables (Koellinger and de Vlaming, 2019). This could bias associations between health outcomes and genetic data. Our inclusion of genetic principal components will address some but potentially not all such biases.

The third possible source of bias arises from cohort effects. Evidence from, for example, American sources (Rosenquist et al., 2015; Walter et al., 2016) indicates an apparently greater effect of SNPs on BMI for individuals born in more recent decades compared to those born in the earlier part of the twentieth century. This phenomenon was attributed in these papers to an increasingly obesogenic environment.

We cannot measure cohort effects separately from age effects since we do not have longitudinal evidence on BMI, which was not collected in Biobank for other than a small subsample of individuals. This means we cannot fully test the association of the allele score with BMI by cohort and by age. There was weak evidence of an association between the allele score and participant age (p = 0.07), and Mendelian Randomization models conditioning on age were similar to those that were not conditional on age. If cohort effects are present, however, then our effect estimates may underestimate the impact of a marginal unit of BMI on hospital costs both for younger cohort members and for individuals that were too young to be recruited into UK Biobank.

### 5.5 Policy relevance

Estimates of the effect of the marginal unit of BMI are relevant to a broad range of policy issues. These issues encompass estimates of the cost-effectiveness of interventions targeting adverse weight profiles, national health systems and by private insurers research priorities, justifications for governmental interventions to target adiposity-related externalities, and for the pricing of insurance policies.

The results of our analysis may be most relevant to policy changes that consider relatively modest changes in BMI, since the amount of variance in BMI explained by the SNPs used is less than 2%, although our evidence that the effect of BMI on hospital costs is approximately linear would allow some extrapolation of the effects of larger changes in BMI.

Any intervention targeting adiposity will appear relatively more cost-effective if the marginal unit of adiposity is higher, and evidence from our analysis suggests that this is plausibly the case. Likewise, a relatively larger “prize” for insurance companies and health systems of reducing BMI (where clinically appropriate to do so) has implications for the prioritization and funding of research (Claxton and Sculpher, 2006; Jackson et al., 2019). Justifications for government intervention, including policies such as higher taxation on sugar-sweetened beverages, are generally motivated – at least in part – by the associated external effects of health-comprising levels of adiposity on various outcomes (Allcott et al., 2019; Cawley and Meyerhoefer, 2012; Cawley et al., 2015b, 2019). Our causal analysis demonstrates both that these effects exist in relation to healthcare cost outcomes, and that they may be larger than those estimated using conventional study designs.

This overall relevance of our estimates depends on two important conditions. The first is the idea of LATE as defined and discussed above. As Basu (Basu, 2011) writes, “LATE is an interpretable parameter when the observed variation in the instrument defines the question for which the analyst seeks an answer, e.g., if the analyst has access to an instrument, Z, that takes two values (z and z′) and the question he seeks to answer is precisely what happens when the instrument is changed from z1 to z′.” This criterion is satisfied in the present case: the unit change in BMI associated with changes in the value of our BMI instrument variable(s) is precisely the type of policy question that we wish to answer. Thus, assuming LATE and the population of compliers is an interesting population, we may be content that the LATE parameter is relevant to policy in this case. Indeed, it is plausible that the effects of BMI in compliers is likely to be similar to the effects of BMI on average across the population, so that LATE is identical to or close to the average treatment effect,

The second issue is that of the stable unit treatment value assumption (SUTVA). This encodes the assumption that the outcome for an individual exposed to a treatment (BMI in this case) is the same irrespective of the mechanism used to assign the treatment (Rubin, 1986). This ensures that the potential outcomes of the treatment are well defined. The original motivation for using random perturbations in genetic variation to identify causal effects reflects this assumption (Davey Smith and Ebrahim, 2003): “The future potential of Mendelian randomization will depend upon the elucidation of functional polymorphisms that mirror environmental exposures of interest.” This reflects the concepts of phenocopy (an “environmental” effect that mirrors genetic variation) and genocopy (genetic variation that mirrors an environmental stimulus) (Ebrahim and Davey Smith, 2008).

Our analysis fails to meet this assumption because we do not know whether (hypothetically) increasing BMI by one unit through manipulating an individual’s diet, exercise regime or environment is precisely identical to increasing BMI through a hypothetical manipulation of their genotype. This distinction can be seen by considering genetic variants in the region of the FTO gene, an important effect of which is to diminish satiety upon eating. As Burgess et al. (2012) note, the consequence of modifying this gene will be to affect food intake by an effect on satiety. This may not have an identical effect to an intervention that reduces BMI by increasing exercise intensity or by an intervention such as bariatric surgery.

The difference in timing of effect between, for example, mid-life interventions targeting BMI and genetically elevated BMI (determined at conception) is another example of how Mendelian Randomization may not satisfy the stable unit treatment value assumption. Mendelian Randomization estimates therefore do not measure the effect of a randomized intervention on the population of interest because our Mendelian Randomization models fail the SUTVA in this case.

We conclude that our preferred estimates (see above) indicate that more conventional study designs underestimate the true causal impact of adiposity, but that the precise magnitude of a genetically predicted change in BMI may not accord with all other possible sources of change in BMI, including those implemented by individual or population-wide interventions. We note that these types of consideration may apply in the same way to most if not all other causal estimates (other than those obtained from well-designed randomized controlled trials) of the association between adiposity and healthcare costs, none of which should be naively interpreted as the effect of randomizing individuals in a population to higher or lower BMI.

Taking these considerations and qualifications into account, we now offer a simple “back of the envelope” illustration of the potential policy impact of our new estimates. If we assume 50 million adults (roughly the size of the adult population in the UK), then using the penalized weighted median Mendelian Randomization estimate of the effect on hospital costs of a marginal unit of BMI (which is intermediate between the high IVW estimate and lower weighted median estimate of £18.85) suggests costs due to one additional unit of BMI as 50,000,000*1*£18.85 = £942,500,000.

Performing the same calculation but using instead the multivariable conditional correlational estimate of £13.47 results in an estimate of additional costs of £673,500,000. This amounts to a difference of £269,000,000 between the estimates for a single year of one unit more of BMI for all adults. One of the many assumptions we make in this simple illustration is that effects obtained using the age profile of adults in UK Biobank (most of whom were aged between 40 and 69 at the time of recruitment) also apply to younger adults.

This figure may be interpreted as the additional costs not available to the hospital system, in one year and amongst all adults, under the higher rather than lower estimates of a single marginal unit of BMI. A population-wide intervention to improve adult BMI may therefore not be cost-effective under traditional estimates, but may be cost-effective under the higher Mendelian Randomization estimates. These types of consideration also apply to decisions to prioritize research targeting BMI compared to other clinical areas.

## 6 Conclusion

We have reported the first Mendelian Randomization analysis to estimate the causal effect of adiposity on inpatient hospital costs. Results suggest that conventional adjusted multivariable analysis probably understates the effect of BMI on hospital costs. Findings from within-family models were imprecise, and we cannot discount the possibility of dynastic biases, although interpretation of these models is complicated by limited power and the possibility of a Type 1 error. Nevertheless, Mendelian Randomization is a feasible and potentially valuable form of analysis for health economics. The methods could be applied in modelling economic outcomes for other traits, behaviours, circumstances and diseases.

## Funding statement

PD, GDS, SH and NMD are members of the MRC Integrative Epidemiology Unit at the University of Bristol which is supported by the Medical Research Council and the University of Bristol (MC_UU_12013/1, MC_UU_12013/9). PD acknowledges support from a Medical Research Council Skills Development Fellowship (MR/P014259/1). The Economics and Social Research Council (ESRC) support NMD via a Future Research Leaders grant [ES/N000757/1]. SH was supported by Health Foundation grant “Social and economic consequences of health status - Causal inference methods and longitudinal, intergenerational data”

## CRediT authorship contribution statement

**Padraig Dixon:** Conceptualization, Formal analysis, Writing - original draft, Writing - review & editing. **William Hollingworth:** Writing - review & editing. **Sean Harrison:** Formal analysis, Writing - review & editing. **Neil M. Davies:** Writing - review & editing. **George Davey Smith:** Writing - review & editing.

## Declaration of Competing Interest

The authors declare no conflicts of interest.

## Footnotes

1 Henceforth we use “multivariable” or “multivariable conditional correlation” as shorthand for all study designs and estimators that do not use either formal randomization or reliance on some kind of natural experiment. We avoid the use of the term “observational” for this purpose, as Mendelian Randomization is itself a form of observational analysis. We also avoid the use of ordinary least squares (OLS) as shorthand for these other study designs as the estimators used were not necessarily of this type.

2 The treatment variable is sometimes referred to as the exposure, the modifiable factor, the risk factor or the intermediate phenotype. Following the econometrics literature, we will refer to BMI as the treatment variable.

3 The estimated effect of a marginal unit BMI differ between those reported in Dixon et al because the sample here is restricted to those with valid genetic data
