# Supplementary material for: Mendelian Randomization analysis of the causal effect of adiposity on hospital costs
Source: J Health Econ. 2020 Mar;70:102300. doi: 10.1016/j.jhealeco.2020.102300 (PMC7188219; doi:10.1016/j.jhealeco.2020.102300)
Supplement: Supplementary file 1 [file mmc1.docx]

**Mendelian Randomization analysis of the causal effect of adiposity on hospital costs**

Padraig Dixon^1,2^*, William Hollingworth^1^, Sean Harrison^1,2^, Neil Davies^1,2^ George Davey Smith^1,2,3^

1: Population Health Sciences, University of Bristol

2: MRC Integrative Epidemiology Unit, University of Bristol

3: NIHR Biomedical Research Centre, University of Bristol

January 2020

_________________________

*Corresponding author. Padraig Dixon, Oakfield House, Oakfield Grove, University of Bristol, Bristol, England, BS8 2BN, padraig.dixon@bristol.ac.uk

# Introduction

This Supplementary Material is arranged as follows:

- Section 2: Creation of elective, non-elective and other cost categories
- Section 3: Explanation of exclusions and participant numbers
- Section 4: Justification for the use of linear models in modelling mean causal effects using Mendelian Randomization
- Section 5: Histogram of body mass index
- Section 6: Graphical summary of results of non-linear analysis
- Section 7: Results of main Mendelian Randomization analyses when conditioning on age

# Creation of elective, non-elective and other cost categories

Elective admissions are those that are planned. Non-elective admissions are not arranged in advance, and may include, for example, maternity care. The “other” category includes all other costs, including day cases, which are elective admissions but where an overnight stay is neither planned nor undertaken. The IVW Mendelian Randomization models were re-run using only costs in each respective category as the outcome variable. We examined effect sizes, p-values and heterogeneity statistics for each model and compared them to the base “all inpatient costs” analysis.

We emphasize that the distinctions between these sub-categories are not absolute, since the categorization is somewhat arbitrary. Elective, non-elective and other costs were calculated as follows.

First, elective, non-elective and other HRGs and their associated unit costs were identified from NHS Reference Costs. Second, these were linked to Finished Consultant Episode (FCE) output obtained from applying the NHS Grouper software to Hospital Episode Statistics data from the UK Biobank cohort. Where the link identified a FCE that associated with a HRG and unit cost that appeared in only one category, then this cost was assigned to that episode.

Second, elective care was identified if the admission method was coded as any of elective admissions from a waiting list, booked, planned, or coded as a transfer from another hospital other than in an emergency. Unit costs were then assigned according to this coding. Some non-elective short stay care was coded as elective care. This was addressed by assigning costs on the basis of the share of elective/non-elective short stay care in total finished consultant bed days reported in NHS Reference Costs. “Other” costs were calculated per individual by subtracting elective and non-elective costs, as calculated above, from total costs.

Results were as follows. Effect sizes for an additional unit of BMI were larger in absolute terms for elective costs than for non-elective costs, and heterogeneity was more pronounced under the former (Cochran’s Q 107.2, p-value = 0.01) than the latter (Cochran’s Q ,p-value = 0.12). These values were both lower than the corresponding value using all inpatient in the main analysis (Cochran’s Q=107.8, p-value=0.01), although the value of the statistic for inpatient-only costs was very similar to that for all costs. Heterogeneity for other costs only was similar to that for non-elective costs (Cochran’s Q=93.9, p-value=0.11).

**Table A1 Disaggregated cost estimates**

|  | **Beta** | **SE** | **P-value** |
| --- | --- | --- | --- |
| **Cost outcome** |  |  |  |
| Base IVW estimates (all costs) | 21.21 | 3.50 | <0.001 |
| Elective costs only | 17.96 | 4.13 | <0.001 |
| Non-elective costs only | 9.00 | 3.80 | 0.02 |
| “Other” costs only | 4.17 | 3.13 | 0.18 |

# Participant numbers

A total of 488,377 cohort participants were successfully genotyped. Analysis was restricted to those self-reporting “White British” ethnicity, or those who had very similar ancestral backgrounds, as determined by principal component analysis. This restricted the sample to 409,703 individuals. Individuals reporting sex mis-match and/or with sex chromosome aneuploidy were excluded (n=814). Kinship was estimated using the KING toolset (1), and identified 107,162 related pairs of individuals. An in-house algorithm removed individuals related to the greatest number of individuals until no related pairs remained, resulting in the exclusion of 79,448 individuals. Additionally, two individuals were related to a very large (more than 200) number of cohort members and were excluded. Finally, 76 individuals withdrew their consent for study participation and their details were removed. After these exclusions, 337,055 individuals remained in the dataset.

The in-house processing of the genetic data is described in more detail in Mitchell et al (2).

Genetic data was also subject to quality controls by UK Biobank (3).

The exclusions for the cost data proceeded as set out in Figure 1 of Dixon et al (4). Valid inpatient hospital cost data and body mass index data were available for 457,689 individuals. Bringing together the genetic and phenotypic (cost, BMI and other data) resulted in the final analysis group comprised of 307,048 individuals.

# Linearity of conventional Mendelian Randomization estimates

Zhao et al (5) demonstrated why linear Mendelian Randomization estimators are likely to be appropriate in a wide variety of circumstances. Our arguments below are identical to those in Zhao, with a slight modification to notation.

The argument proceeds as follows. The estimand of interest is the effect of treatment (BMI ) on the outcome (costs), as mediated by the instrumental variable G and conditional on omitted variables U. Without loss of generality, assume G is binary, and takes the value of 1 (“instrument is switched on and influencing the treatment variable”) or 0 (“instrument is switched off and is not influencing the treatment variable”). This is the conventional definition uses to estimate treatment effects in instrumental variable analysis:

$$E\left[ h\left( Y \right) | G_{i}=1 \right]-E\left[ h\left( Y \right) | G_{i}=0 \right]$$

This can be equivalent to a more general formulation of the same relation as follows, following the Zhao et al terminology

$$E\left[ h\left( Y \right) | G_{i}=1 \right]-E\left[ h\left( Y \right) | G_{i}=0 \right]=E[h(f\left( X\left( 1 \right),U,\varepsilon_{y} \right)-h(f\left( X\left( 0 \right),U,\varepsilon_{y} \right)]$$

Here, X(1) is the value of BMI when the instrument takes the value 1, and likewise for X(0)).

Now approximate this function using the first term of a Taylor series expansion, using the fact that the difference between $X\left( 1 \right)$ and $X\left( 0 \right)$ will be small because individual SNPs have modest impacts on treatment variables:

$$E[h'(f^{1}\left( X,U,\varepsilon_{y} \right)*\left( X\left( 1 \right)-X\left( 0 \right) \right)]$$

In this expression, $h'$ is the derivative of the arbitrary $h$ function and $f^{1}$ is the first derivative of the function relating outcomes to BMI, omitted variables and the random noise term. The final step is to recognise that the variance of the outcome Y is much bigger than the casual effect of BMI on the outcome, which means that we can take expectations over each product.

$$E[h'(f^{1}\left( X,U,\varepsilon_{y} \right)]*E[\left( X\left( 1 \right)-X\left( 0 \right) \right)]$$

This expression is approximately equivalent to our first equation above:

$$E\left[ h\left( Y \right) | G_{i}=1 \right]-E\left[ h\left( Y \right) | G_{i}=0 \right]\approx E[h'(f^{1}\left( X,U,\varepsilon_{y} \right)]*E[\left( X\left( 1 \right)-X\left( 0 \right) \right)]$$

The left-hand side of this equation is the causal effect on the outcome when the value of the instrumental variable differs; this is simply $\Gamma_{j}$ from the main paper – the effect of SNPs on the outcome. The last term on the right-hand side of the equation is the effect on BMI of the SNPs – this is $\gamma_{j}$. The equation we have defined therefore entails a linear relationship between these two terms:

$$\Gamma_{j}={\beta\gamma}_{j},$$

Where $\beta= E[h'(f^{1}\left( X,U,\varepsilon_{y} \right)]$. The key issue is that, whatever the nature of the h or f functions involved, our interest if defined solely by this relationship between $\Gamma_{j}$ and $\gamma_{j}$. This linearity will hold even if these functions are elsewhere non-differentiable.

# Distribution of body mass index

Figure A2 summarises the baseline distribution of body mass index in the analysis sample.

**Figure A2 Histogram of body mass index**

# Graphical summary of non-linear analysis

Figure A3 is a graphic summary of the non-linear analysis. Note that results are reported relative to mean BMI – this is because the function is based on local average causal effects in one hundred quantiles and does not include an intercept. The value of the function at a particular point represented the expected difference in costs when BMI is at a particular level compared to BMI at the reference level of mean BMI, which is approximately 27 kg/m^2^. The outer lines are 95% confidence intervals.

**Figure A3 Fractional polynomial non-linear Mendelian Randomization model**

# Results of main Mendelian Randomization estimates conditioning on age

Table A4 below summarises Mendelian Randomization estimates for the main models but additionally conditioning on the age of participants at baseline.

**Table A4 Results of primary Mendelian Randomization models conditioning on age**

|  | **Beta (£)** | **SE** | **P-value** |
| --- | --- | --- | --- |
| **Estimator** |  |  |  |
| IVW RE | 21.94 | 3.47 | <0.001 |
| MR-Egger | 6.55 | 8.32 | 0.43 |
| Penalized weighted median | 18.29 | 4.95 | <0.001 |
| Weighted mode | 15.23 | 6.30 | 0.02 |

Cochran’s Q reveals some evidence of heterogeneity for the IVW estimates (Q=108.01, p-value=0.01).

References

1. Manichaikul A, Mychaleckyj JC, Rich SS, Daly K, Sale M, Chen W-M. Robust relationship inference in genome-wide association studies. Bioinformatics (Oxford, England). 2010;26(22):2867-73.

2. Mitchell R, Hemani G, Dudding T, Paternoster L. UK Biobank Genetic Data: MRC-IEU Quality Control, Version 1. University of Bristol; 2017.

3. Bycroft C, Freeman C, Petkova D, Band G, Elliott LT, Sharp K, et al. The UK Biobank resource with deep phenotyping and genomic data. Nature. 2018;562(7726):203-9.

4. Dixon P, Davey Smith G, Hollingworth W. The Association Between Adiposity and Inpatient Hospital Costs in the UK Biobank Cohort. Appl Health Econ Health Policy. 2018.

5. Zhao Q, Wang J, Bowden J, Small DS. Statistical inference in two-sample summary-data Mendelian randomization using robust adjusted profile score. arXiv preprint arXiv:180109652. 2018.
